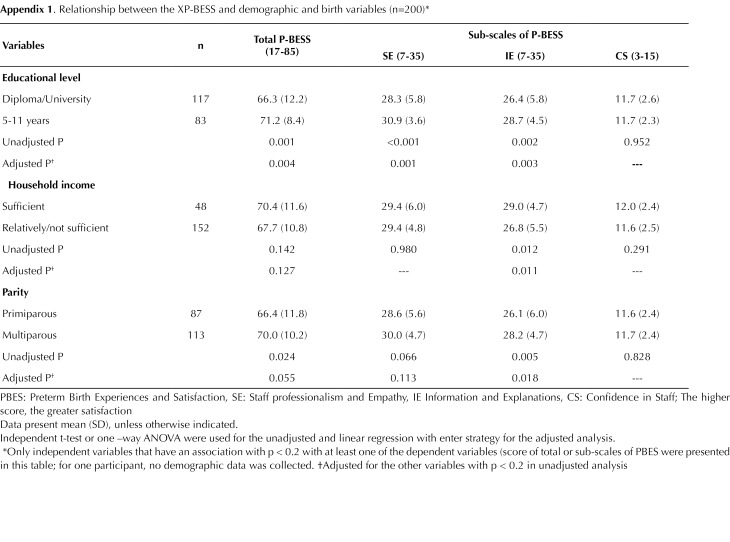# Validity and reliability of the Iranian preterm birth experiences and satisfaction scale: A methodological and cross-sectional study

**DOI:** 10.34172/hpp.2021.13

**Published:** 2021-02-07

**Authors:** Maryam Najjarzadeh, Sakineh Mohammad-Alizadeh-Charandabi, Shamsi Abbas-Alizadeh, Mohammad Asghari Jafarabadi, Mojgan Mirghafourvand, Nahid Tagipour-Amidi, Alexandra Sawyer, Susan Ayers

**Affiliations:** ^1^Student Research Committee, Faculty of Nursing and Midwifery, Tabriz University of Medical Sciences, Tabriz, Iran; ^2^Social Determinants of Health Research Center, Department of Midwifery, Faculty of Nursing and Midwifery, Tabriz University of Medical Sciences, Tabriz, Iran; ^3^Women’s Reproductive Health Research Center, Alzahra Hospital, Tabriz University of Medical Sciences, Tabriz, Iran; ^4^Road Traffic Injury Research Center, Department of Statistics and Epidemiology, Faculty of Health, Tabriz University of Medical Sciences, Tabriz, Iran; ^5^Department of Midwifery, Faculty of Nursing and Midwifery, Tabriz University of Medical Sciences, Tabriz, Iran; ^6^School of Health Sciences, University of Brighton, Brighton, UK; ^7^Centre for Maternal and Child Health Research, School of Health Sciences, City, University of London, UK

**Keywords:** Patient satisfaction, Premature birth, Psychometrics, Validation study, Iran

## Abstract

**Background:** Women’s experience and satisfaction with childbirth care are increasingly being addressed by health care providers, managers, and policymakers. Due to need for a validated special scale for assessing satisfaction of women with preterm birth, we aimed to translate and adapt the first specific tool, Preterm Birth Experiences and Satisfaction Scale (P-BESS), into Persian language and evaluate its psychometric properties.

**Methods:** A methodological and cross-sectional study was conducted in two tertiary levels of maternal hospitals in Tabriz. The Iranian version of the scale was developed from the original English version using forward-backward translation. After confirmation of its face and content validity, the scale was completed by 201 women who had given birth prematurely.

**Results:** Exploratory factor analysis revealed three factors (staff professionalism and empathy, confidence in Staff, information and explanations) with eigenvalues greater than 1, explaining a total variance of 55.4%. Confirmatory factory analysis showed that the 17-item, three factor model fitted the data well: the root mean square error of approximation 0.060. There were moderate correlations between the total and sub-scales of Iranian P-BESS and overall satisfaction (r = 0.45 to 0.66), as well as need for improvement (r = -0.46 to -0.61), which confirm convergent validity. Internal consistency and test–retest reliability of the scale and its sub-scales were satisfactory (α = 0.852 to 0.922, intraclass correlation coefficient; 0.83 to 0.92).

**Conclusion:** The Iranian version of P-BESS is a valid and reliable scale which can be used by policy makers, managers, health care providers and researchers.

## Introduction

Preterm birth accounts for 11% of all live births worldwide^1^ and 10% in Iran.^2,3^ It is the most common cause of neonatal mortality. About three quarters (76%) of neonatal mortality in Iran occurs among those infants with low birth weight (< 2500 g). The neonatal mortality rate is about 10 per thousand of all live births and 80 per thousand of those with low birth weight.^4^

The process of onset and occurrence of preterm labor is often rapid and unexpected, and the newborn baby is often immediately separated from the mother and placed in a specialized care unit, which makes preterm labor a stressful experience for parents.^5,6^ Mothers facing preterm birth usually have different emotional reactions compared to those with full-term birth; their stress and anxiety is the origin of psychological trauma which can lead to post-traumatic stress disorder and affect on the mother-infant interaction.^7^

Women’s experiences of childbirth are a combination of related physiological and psychological events and are considered as important childbirth outcomes.^8^ Women’s childbirth experiences stay with them for a long time.^9^ Negative childbirth experiences also affect mothers’ future fertility behaviors; these mothers become pregnant again after a longer period, and give birth to fewer children.^10^

Women’s views, experiences, and satisfaction with maternal care -especially childbirth care- are increasingly being addressed by health care providers, managers, and policymakers and can influence the organization’s decisions and services provided.^11,12^ Organizations that place greater emphasis on improving the experiences of their clients have better care outcomes.^13^ Satisfaction measurement is a way of assessing the care process, describing the views of clients, and evaluating care.^12^ Client satisfaction is a major outcome of the interaction between service providers and clients, which is a measure of the quality of care provided.^14^

Questionnaires are the most common tools for measuring satisfaction. Many general tools have been developed to measure satisfaction during labor and childbirth. However, these tools cannot adequately measure the satisfaction of parents with high risk infants,^15^ because this particular group of parents reports different experiences.^6^ Due to the lack of specific tools for assessing the satisfaction of parents with preterm infants, Sawyer et al designed and evaluated validity and reliability of Preterm Birth Experiences and Satisfaction Scale (P-BESS) in the UK in 2014, using the results of their qualitative study on the experiences of parents with preterm infants, review of the literature, and discussions with related experts.^16^

The questionnaire has three domains: (1) staff professionalism and empathy referring to professional competencies and staff’s warm and friendly attitude, (2) confidence in staff demonstrating capability of staff to control the situation, and (3) information and explanations, i.e. participants tend to be told what would happen during the childbirth.^6^ These domains are considered as important aspects of satisfaction with health care.^17^

Researchers and physicians can use the total score or subscale scores depending on their needs. The total score can compare different hospitals or clinical care while individual aspects of the care setting can be compared via the subscales.^16^ Further evaluation of its psychometric properties in different population has been recommended by the devdelopers,^16^also in the review of scales for measuring women’s childbirth experiences.^18^ The validity and reliability of its Spanish version have also been confirmed.^19^

Considering the need for a validated special scale for assessing satisfaction of women with preterm birth and lack of such a scale in the country, we aimed to translate, adapt the first specific tool, P-BESS, into Persian language and evaluate itspsychometric properties.

## Materials and Methods

### Design and aims

This is a methodological cross-sectional study. Aims of this study were to translate and cross-culturally adapt the original UK version of P-BESS into Persian language, and to evaluate its psychometrics properties in a group of Iranian women with preterm birth.

### Participants, sampling and settings

Participants were women who had given birth prematurely (gestational age of less than 37 weeks) within the previous 24 to 72 hours and their newborns were alive. Those who were not able to read and write Persian fluently, were excluded.

The participants were recruited using proportional stratified sampling (stratified by hospital) from Alzahra and Taleghani hospital, the only tertiary level maternal hospitals in East Azerbaijan province. Almost allpotentiallyeligible women were approached during the study periods (except some days that the investigators were not able to attend the study settings). All approached women who were eligible and willing to participate were included into the study.

East Azerbaijan province has about 4 000 000 population with 61.5 thousand births annually.^20^ Taleghani hospital is a tertiary hospital for referral from other centers in provincial cities. Alzahra hospital is a tertiary hospital for referral from other centres in provincial cities, and also from two nearby provinces. According to the national guidelines, providers working at primary and secondary level hospitals are required to refer all pregnant women with gestational age of less than 34 weeks who have symptoms of preterm labor to tertiary maternal level hospitals.^21^

At the study centers, midwives at the birth ward work with case method, i.e. any midwife at each working shift is responsible for one to two special hospitalized women. For preterm labors, they are required to monitor the women’s fetal heart rate, vital signs and labor progress with minimal interventions. At the moment of a preterm childbirth, in addition to the birth attendant, a person trained in neonatal resuscitation (usually an experienced midwife) also presents at the delivery room. In the case of births less than 32 weeks gestation or when the need for neonatal resuscitation is high, a neonatology or pediatric resident is also present at the birth. A study conducted between 2013 and 2014 reported that the survival rate of infants with birth weight ≤ 1500 g or gestational age ≤ 32 weeks until discharge from the hospitals was 71%.^22^

### Sample size

For factor analysis, recommended sample size is at least five to ten participants per item.^23^ For a 17-item measure, 85 is sufficient for exploratory factor analysis (EFA) and about 85+ is sufficient for confirmatory factor analysis (CFA).^24^

### Instrument translation

We obtained permission from developers of the P-BESS to translate and validate the scale in Persian. The 17-item P-BESS is scored using a five-point Likert scale (5 = strongly agree, 4 = agree, 3 = neither agree nor disagree, 2 = disagree, 1 = strongly disagree), with four items reverse scored (items 10, 12, 14, 16). The original scale consists of three sub-scales: Staff Professionalism and Empathy (SE, 7 items), Information and Explanations (IE, 7 items), and Confidence in Staff (CS, 3 items). Total and sub-scales scoring are calculated by sum score of the related items. The original scale includes two additional items for evaluation of partner involvement when the woman’s partner attended the birth.^16^ These items were omitted from the study, because in the study settings a woman’s partner is not permitted to attend the birth.

We used the four-step translation guideline recommended by Guillemin et al to create the Iranian P-BESS; (i) translation, (ii) back translation, (iii) panel review of those translations, and (iv) pre-testing.^25^ The UK P-BESS^16^ was first independently translated into Persian language by two female Iranian healthcare professionals, emphasizing conceptual not literal equivalence. The translators were at a proficient level in English and were experts in midwifery. Then, the translators compared their translations and discussed and agreed on the first version of the translated Iranian scale. The first version was then back translated into English by an English native female person who was proficient in Persian language, was not an expert in midwifery and had no knowledge of the original scale. The original scale and the back translated scale were then compared and discussed in a panel of the Iranian research team. This discussion led to only several minor modifications in the wording of items. This translated version alongside with the original UK version of the items, were then given to 10 experts to get their opinion about their translation. The experts were academic members at Tabriz University of Medical Sciences in different specialisms; three in midwifery, two in reproductive health, one in maternal nursing, three obstetricians and gynecologists, and one biostatistician. Minor revisions in wording were made based on the experts’ comments. A pre-test using cognitive interview was then conducted with 15 women who had study eligibility to ensure comprehensibility and legibility of the items. During these interviews women were asked to explain their thoughts on the meaning of each item. All items were found to be acceptable and understandable for all of the women. Only a very minor revision was carried out and the final version named the Iranian P-BESS.

### Content validity (including face validity)

To calculate the degree to which the content of the scale is an adequate reflection of the construct to be measured,^26^ the above-mentioned experts commented on necessity (“essential”, “useful, but not essential”, or “not necessary”) and relevancy (“not relevant”, “somewhat relevant”, “quite relevant”, “highly relevant”) of each item. Content validity ratio (CVR) was calculated using the formula developed by Lawshe (i.e. (ne- N/2)/(N/2), ne = number of experts with necessity rating of essential, N = number of all experts). According to the Lawshe’s tabulation a CVR ≥ 0.62 was considered as satisfactory.^27^ Content validity index (CVI), the proportion of experts with relevance rating of quite or highly relevant, greater than 0.79 was considered reasonable.^28^ We also placed an open-ended question for each item to illicit opinion of the experts concerning meaning.^29^

According to the COnsensus-based Standards for the selection of health status Measurement INstruments (COSMIN) study, face validity is an aspect of content validity.^26^ For evaluation of face validity, we used quantitative and qualitative methods. During the qualitative evaluation, 15 eligible women were asked about levels of difficulty, irrelevancy and ambiguity of each item. Based on their opinion, a minor revision was done on the scale. Also, in the quantitative evaluation, the importance of each item was determined by the women, as well as the 10 experts using a 4-point Likert scale ranging from 1 (not important at all) to 4 (very important). An impact score was calculated using the formula of Frequency × Importance. Frequency reflects proportion of respondents with importance rating of 4 and importance reflects the mean score. An impact score of greater than 1.5 for each item was considered acceptable.^30^

### Floor and ceiling effect 

We also calculated floor or ceiling effect, which shows frequency of lowest or highest possible score achieved by respondents. If 1%-15% of respondents choice lowest or highest possible score, there is small effect and met standards.^31^

### Construct validity

At first, the factor structure of the P-BESS was extracted using the EFA. Then, we conducted a CFA to assess how well the EFA extracted model fit the observed data.

### Convergent validity

This validity refers to the degree to which a new scale is related to other measures of the same construct.^32^ As suggested by the scale developers, it was explored through calculating correlations between total and sub-scale scores of P-BESS and the following two items with a five-point Likert score; “I was very satisfied with the care during the birth” and “The care during the birth could have been improved”.^16^

### Divergent/discriminant validity

This validity refers to lack of significant correlation of the construct with unrelated variables.^33^ According to the results of our previous study at the same setting^34^ and other previous studies in Iran,^35^ and in other countries using other satisfaction scales,^36,37^ we predicted no statistically significant correlations between total score of Iranian P-BESS, also between scores of its sub-scales and age.

### Reliability

Both internal consistency (the degree of the interrelatedness among the items^26^) and test-retest reliability (resistance of measurement score to change over time^26^) were evaluated. Test-retest reliability was evaluated by completing the scale twice by 20 women with a two week interval. Also, to determine consistency of any item with the average behavior of the other items, an item-total correlation was calculated.

### Data collection

Participant recruitment and data collection were conducted during morning or afternoon shifts by the trained investigators (MN or NTA) who had interview experience in similar studies. During the study period, the investigators identified potentially eligible women daily from the online national birth registration system. Then they attended the postnatal wards to approach the potentially eligible women. After assessing eligibility criteria and obtaining informed written consent, all questionnaires were completed in the presence of the investigators at a private place in the postnatal ward. The P-BESS was self-administered but the other parts of the questionnaire such as demographic and obstetric data was completed through interview. The investigators had no involvement in care of the woman or her baby.

### Data analysis 

Normal distribution of quantitative variables was assessed using skew and kurtosis, and -2 to +2 for skew and -7 to +7 for kurtosis were considered acceptable for the normality.^38^ The data was described using mean, standard deviation (SD) and range for quantitative variables, with normal distribution and number (percent) for categorical variables.

Since the main purpose of the study was to extract the dimensions and their inter-relations, we used covariance based EFA and CFA and not partial least squares based CFA.^24^ Principal axis factoring using oblimin with Kaiser normalization rotation method was used for the factor extraction in the EFA. Value > 0.7 in the Kaiser-Meyer-Olkin (KMO) test was considered acceptable for the factor analysis, and *P* < 0.050 in the Bartlett’s test was considered data fit for detectable relations between variables that were to be factor analyzed.^39^ Factor-item loading values ≥ 0.40 were considered as satisfactory for allocation of an item to the factor. CFA was conducted using maximum-Likelihood estimation. Root mean square error of approximation (RMSEA) less than 0.08, and goodness of fit index (GFI), comparative fit index (CFI), normed fit index (NFI) and incremental fit index (IFI) greater than 0.90 was considered as an adequate model fit.^40^

Cronbach’s alpha coefficient was used to assess internal consistency of the total and sub-scales of the Iranian P-BESS data and a threshold of 0.7 or greater was considered satisfactory.^40^ Item-total correlation of less than 0.30 was considered unsatisfactory. Test-retest reliability was calculated using intraclass correlation coefficient (ICC) and its 95% confidence interval (CI), and values of 0.74 to 0.82 was considered good and greater than 0.90 was considered very good reliability.^41^

The Pearson test was used to determine correlations between the scale scores and women’s age, and independent *t* test or one-way analysis of variance (ANOVA) were used to determine unadjusted associations between the scale scores and categorical variables and linear regression to determine the adjusted relations (adjusted for variables with *P* < 0.2 in the unadjusted analysis).

Statistical analysis was conducted using Statistical Package for Social Sciences (SPSS) version 21 (IBM, Chicago, IL, USA) and the Analysis of a MOment Structures (AMOS) version 18. *P* values < 0.05 were considered to be significant.

## Results

Data was collected between August 2019 and December 2019. Out of 243 women approached, 201 were eligible and included the study. 42 women were excluded due to low literacy levels. Mean time to complete the P-BESS was 12 minutes. All included women completed all questionnaires, except one who did not complete the questionnaire related to demographic and obstetric characteristics.

Approximately three-quarters (77%) of the participants had an education of 12 years or less, about half (44%) were primiparous and two-thirds (66%) had cesarean section. Mean age of the participants was 29.3 (SD = 6.9) years and mean gestational age was 33.3 (SD = 2.4) weeks. Other demographic and obstetric characteristics are shown in Table 1.

### P-BESS Characteristics

Tables 2 and 3 show descriptive characteristics of the individual items, the three dimensions and total score of P-BESS which were normally distributed. Mean percentage of the total possible P-BESS score was 75.6 (SD 16.2) and for the sub-scales were between 72.3 and 79.9. The P-BESS total score was strongly correlated with the SE (r = 0.91), IE (r = 0.88) and CS (r = 0.67) sub-scales’ score. There was a moderate correlation between scores of IE with SE (r = 0.66) and CS (r = 0.40), as well as between SE and CS (r = 0.55). These correlations were all significant.

Frequency of possible minimum percent (floor effect) was 0.5% for the total and all three domain scores, and the possible maximum percent (ceiling effect) was 4% for the total score; 20% for SE, 10.5% for IE, and 12% for CS scores.

### Content and face validity

In the qualitative evaluation, according to the experts’ and women’s comments, almost all of the items were grammatically correct and the words were suitable. In the quantitative evaluation, the CVR was satisfactory (ranged 0.73 to 1.0), and CVI was reasonable (ranged 0.76 to 1.0) for all of the items. The least item impact score was 3.6. Therefore, no items were excluded from the scale.

### Explanatory factor analysis

All of the 17 items were entered in the factor analysis. The KMO test with value of 0.910 and Bartlett’s sphericity test with *P* < 0.001 confirmed the data fit for the factor analysis. Three factors with eigenvalues greater than 1 were revealed, explaining a total variance of 55.4%. Factor 1 (staff professionalism and empathy) accounted for 42.9% of the variance, factor 2 (confidence in staff) for 8.0% and factor 3 (information and explanation) for 4.5%. Table 4 shows factor weights of the scale items.

### Confirmatory factor analysis

**Figure 1:**
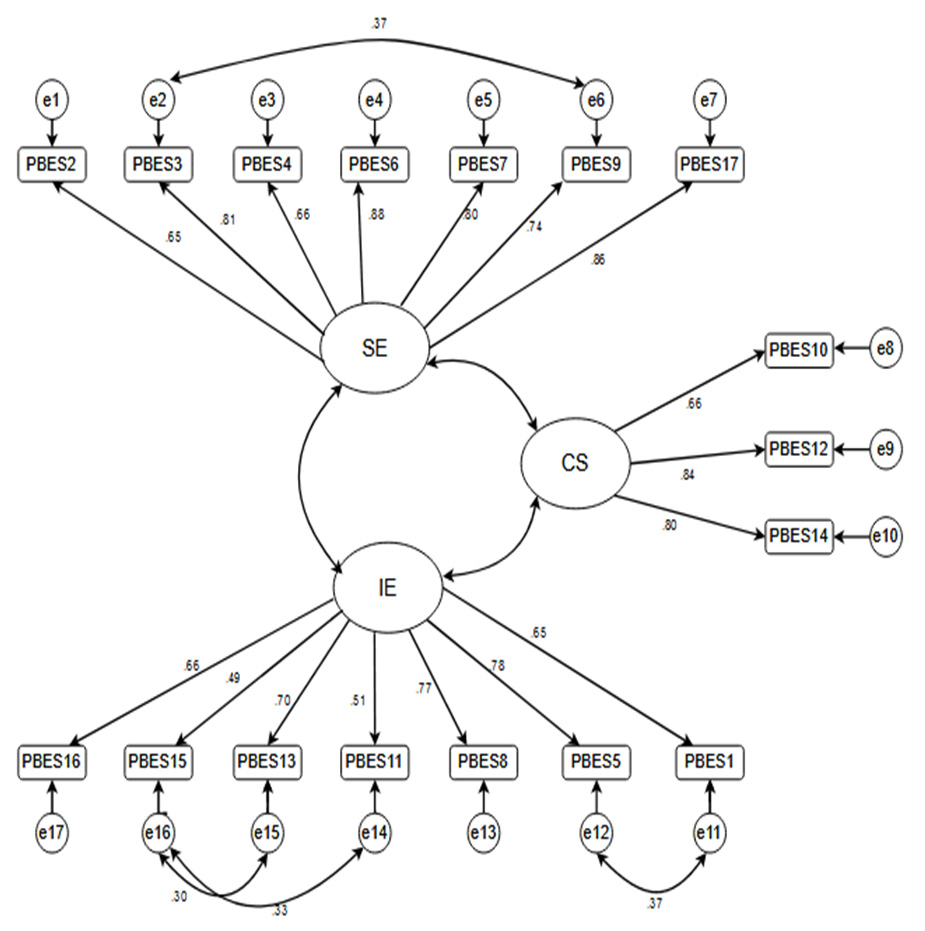
Measurement model of Iranian preterm birth experiences and satisfaction scale extracted from confirmatory factor analysis. Empathy: Staff professionalism and Empathy, Information: Information and Explanations, Confidence: Confidence in Staff; details of the items can be found at table 2; the figures near the arrows from the factors to the items presents the standardized regression coefficient weight of the factor on the indicator; the double headed arrows between the factors indicates the covariance between factors.

The initial model had not good fit (RMSEA = 0.089, 90% CI 0.075 to 0.103; GFI = 0.807; CFI = 0.875; NFI = 0.840; IFI = 0.875). Hence based on modification indices, we add the suggested path that had theoretical interpretations into the model. In the final model, there was a good fit to the three-factor measurement model of the P-BESS (RMSEA = 0.060, 90% CI 0.046 to 0.74; GFI = 0.907; CFI = 0.957; NFI = 0.904; IFI = 0.957). Figure 1 shows the three factors, 17-item measurement model of the Iranian P-BESS.

### Convergent validity

There was a moderate positive correlation between the one-item “overall satisfaction” and total P-BESS (r = 0.656), as well as all its subscales (r = 0.602 for SE, 0.567 for IE, 0.446 for CS). Also, there was a moderate negative correlation between the one-item “need for improvement” and total P-BESS (r = -0.606), as well as all its subscales (r = 0.547 for SE, 0.511 for IE, 0.461 for CS). All of the correlations were statistically significant (*P* < 0.001).

### Divergent validity 

There was no statistically significant correlation between woman age and the total score of the Iranian P-BESS (r = -0.048, *P* = 0.508) and its sub-scale scores (r = -0.005, *P* = 0.947 for SE; r = -0.072, *P* = 0.314 for IE, and r = -0.067, *P* = 0.347 for CS).

### Reliability of the P-BESS

Internal consistency for the total scale (α = 0.922) and its sub-scales (α was 0.911 for SE, 0.852 for IE and 0.808 for CS) was high. All individual items correlated well with the total scale (r ranged from 0.556 to 0.772). There was also a good to excellent agreement between test and re-test results in terms of the total score (ICC 0.88, 95% CI 0.63 to 0.96) and its sub-scales (ICC for SE 0.85, 95% CI 0.55 to 0.95; IE 0.83, 95% CI 0.50 to 0.94; CS 0.92, 95% CI 0.76 to 0.97).

### Relationship between the P-BESS and demographic and birth variables

There were no significant correlations between the total score, as well as the domains’ scores, and the majority of the demographic and birth characteristics including type of childbirth (vaginal birth/emergency cesarean section/repeated cesarean section), child sex, attendance at childbirth preparation classes (yes/no), gestational age at birth (<32^0^/ 32^0^-33^6^/ ≥ 34^0^), intended pregnancy (yes/no), skin to skin contact at the first hour after childbirth (yes/no), breastfeeding at the first day after childbirth (yes/no).

In unadjusted analysis, women with diploma/university education compared to those with less than 12 years education had significantly lower satisfaction in the total (66.3 vs 71.2, *P* = 0.001), also in the SE (28.3 vs 30.9, *P* < 0.001) and IE (26.4 vs 28.7, *P* = 0.002) domains but not in the CS domain (11.7 vs 11.7, *P* = 0.952). Women with sufficient household income compared to those with relatively/not sufficient income had significantly greater satisfaction only in the IE domain (29.0 vs 26.8, *P* = 0.012). Primiparous compared to multiparous women had significantly lower scores in the total (66.4 vs 70.0, *P* = 0.024) and in the IE (26.1 vs 28.2, *P* = 0.005) domain but not in the SE and CS domains.

In adjusted analysis, diploma/university education had significant association with low satisfaction in the total (*P* = 0.004), also in the SE (*P* = 0.001) and IE (*P* = 0.003) domains, and relatively/not sufficient income and primiparous had significant association with low satisfaction only in the IE domains (*P* = 0.011 and *P* = 0.018, respectively) (Appendix 1).

## Discussion

In our best of knowledge, this is the first study evaluating psychometric properties of the first specific tool for assessing satisfaction of women with preterm birth in a country outside Europe. The findings indicate that the three-factor structure of Iranian P-BESS with 17 items is a valid and reliable tool to evaluate birth experience and satisfaction in an Iranian population of women with preterm birth. The findings are generally consistent with those of the original UK^16^ and the Spanish^19^ P-BESS versions.

No need for exclusion or substantial change in the main items of the scale illustrates its simplicity and transferability in different cultures. Elimination of the fourth domain of the scale “partner involvement” which included two items can be considered as part of the cross-cultural adaptation of the tool.

Since P-BESS is a self-report/read and write tool, we excluded illiterate and less literate women, those who could not easily read, and write Persian. Therefore, in terms of translation into Persian language and adaptation, there is no concern that this study was conducted in Tabriz with the participation of Azerbaijani speaking women. If the scale was completed through interview with the women, the interviewer sometimes might have had to state the items in language of Azerbaijani, especially when interviewing less literate or illiterate women, and in that case some problems could arise in transferring the concepts, creating difficulties in adaptation of the tool in Iranian context.

Mean percentage of the total possible score in this study (75.7) was only slightly lower than those reported in the UK study (77.2),^16^ but it was significantly lower than those reported in the Spanish study (80.6).^42^ Similar difference has been reported in the mean score of childbirth satisfaction of women with full-term birth in Iran (58.6) and Spain (70.1).^34,37^ The difference can be attributed at least in some part to use of shared decision making (SDM). Reviews indicate SDM can promote patient satisfaction.^43,44^ In Spain’s health system, it is more than two decades that there has been a growing recognition of the importance of using SDM,^45^ but in the Iran’s health system, SDM are not usually used and initiatives to promote it are in their infancy.^46^

As P-BESS is the first scale assessing experience and satisfaction of women with preterm birth, we found no other similar study in Iran in this area. However, comparison of mean percentage of the total possible score of this study with that of the previous study^34^ in this setting on women with full-term pregnancy indicates higher satisfaction of participants in this study (75.5 vs 60). Similar difference has also been reported in the Spanish studies on women with preterm birth^42^ and those with full-term birth (85.5 vs 70.1).^37^ One possible reason for such a difference could be short duration of labor and less medical interventions such as labor induction, labor augmentation, and excessive vaginal exam in women with preterm births. Studies indicate predictive effect of labor dystocia, and the medical interventions on low birth satisfaction score^47^ and traumatic birth experience.^48^

Similar with the previous studies,^16,19^ the factor analysis in our study indicates that P-BESS is a multidimensional scale. The strong correlation of the P-BESS total with its sub-scales, and moderate correlation between the subs-scales indicates divergent validity between similar, yet theoretically separate constructs represented by the sub-scales. The three factor structure of Iranian P-BESS and the items loaded within the factors were consistent with the UK version of the scale.^16^ Although the Spanish version^19^ also showed a three factor structure, the item “There were occasions when no one explained to me what was going on” loaded in the other factor “Confidence in Staff”.^16^

The EFA results were confirmed by CFA using the model fits statistics such as RMSEA, CFI and NFI which are universally accepted methods for the confirmation.^49^ These results are consistent with the Spanish study,^19^ but results of CFA were not reported in the UK’s study.^16^

The two items (“overall satisfaction” and “need for improvement”) which were suggested by the scale developers to be used to explore convergent validity were entered in the EFA in the Spanish study. The first one loaded in the subscale of “staff professionalism and empathy” and the second one in subscale of “information and explanations”.^16^ However, similar with the UK version^16^ we did not enter them in the EFA. The moderate correlations between each of these two items and total and sub-scales of P-BESS indicate evidence of convergent validity of the scale. Similar with the study in UK,^16^ the highest strength of correlation was between the item of overall satisfaction and total P-BESS.

As expected, in line with the study in Spain,^19^ correlation between the “staff professionalism and empathy” and “information and explanations” sub-scales was higher and correlation between the “confidence in Staff” and “information and explanations” sub-scales was lower than correlations between the other sub-scales.

Similar with the UK study,^16^ the subscale “staff professionalism and empathy” explained the largest proportion of total variance in our study; although the percentage explained by this subscale in this study was slightly lower than the UK study (43% vs 51%). Women with preterm birth are mostly concerned about their infant health,^7^ therefore staff professionalism and empathy could have highest importance for them.

In our study, the factor of “information and explanations” explained the smallest proportion of total variance, but this factor explained largest proportion of total variance in the Spanish study.^19^ The difference in use of SDM in national health systems of the countries may explain the reason for such differences. Because, the factor of information and explanations, patient-health professional communication and describing options are essential for the SDM.^50^

Satisfactory internal consistency for the total scale and subscales in our study is consistent with findings of the studies conducted in UK^16^ and Spain^19^ and the good to excellent agreement between test and re-test results is consistent with the Spanish study but ICC was not reported in the UK study.

Our results about higher satisfaction score of women with low educational level is consistent with results of the Spanish^42^ and some other studies.^51,52^ In the scientific literature, dissatisfaction is defined as a gap between client expectations and the experience of the service.^53^ As the patients education increases, their expectation of the medical care rises.^54^ Therefore, the low satisfaction scores in educated women are probably consequence of their higher expectation from maternity care services.

### Strengths and limitations 

Validation of Iranian P-BESS using various validity and reliability indexes on all women with preterm birth and not limiting the study population to only extremely or very preterm birth can be considered as strengths of this study. The original scale was developed using a population of women with under 32 week’s childbirth. But, similar with the study in Spain,^19^ we conducted this validation study on all women with preterm birth, and our findings showed no significant differences in satisfaction across those women who gave birth at under 32, 32-34 and over 34 weeks’ gestation. Globally, more than 80% of preterm births occur between 32^0^-36^6^ weeks gestation.^1^ Therefore, the scale could be used in a wider population of women with preterm birth.

Although this study was conducted in two teaching hospitals affiliated with Tabriz University of Medical Sciences, its results are likely to be generalizable to the entire province and nationally. This is because most of the premature births, especially births before 34 weeks in the province, and even in some cases from the nearby provinces, are carried out in these hospitals, especially the Alzahra hospital; where about three-quarters of the sample was taken from.

Like the Spanish version,^19^ the questionnaires in this study were completed 24-72 hours after childbirth, before discharge. Although some researchers believe that the time of completing the questionnaire may affect the results of the satisfaction assessment, there is no sufficient evidence to support such a conclusion.^55^ Developers of the original tool collected data via post from the women giving birth over the past year, but they reported that the interval between childbirth and completion of the scale did not affect the overall and subscale scores.^16^

Mothers of premature infants are physically and psychologically vulnerable during hospitalization, and this may affect their care satisfaction assessment.^8,56^ Also, since the staff is expected to provide long-term care for some preterm infants, the mothers may not be comfortable in assessing satisfaction with care,^7,16^ or may not express their true views due to the halo effect.^57^ In this study, to overcome these potential problems, we completed all questionnaires outside the neonatal intensive care units, in an environment away from the staff, and emphasized to the participants about confidentiality. The good to excellent agreement between test and re-test results in present study, as well as in the study in Spain^19^ also confirm that there is no significant difference in satisfaction scores before and after discharge. The results of a previous study in the same study setting on newly delivered women using Iranian-Persian version of Birth Satisfaction Scale Revised (IP-BSS-R) also showed that the satisfaction score assessed 12-24 hours after childbirth (before discharge) is only negligibly higher than the score assessed about 45 days after childbirth.^34^ However, further studies on women with preterm births are recommended at different time points before and after discharge of their infants to assess the effect of the halo effect on the satisfaction.

## Conclusion

The Iranian version of P-BESS, the first specific tool for assessing experience and satisfaction of women during preterm birth, is a valid and reliable scale. Assessment of women’s views, experiences, and satisfaction with childbirth are very important for improving care outcomes, and a specific valid scale are needed for assessment of pre-term birth experience and satisfaction. Therefore, policy makers, managers and health care providers can use the scale to identify areas for improvement of the services for women with preterm birth. Also, researchers can use it as a valid measure in related studies.

## Acknowledgments

We would like to thank authorities of the University for the scientific and ethical approval and financial support of this research. We also sincerely thank all participating women, authorities and personnel of Alzahra and Taleghani hospitals for their cooperation in this study.

## Funding

This work was supported by the Research Vice-Chancellor of Tabriz University of Medical Sciences in Iran. The funding center had no role in the design, analysis or writing of this paper.

## Competing interests

We declare that there is no competing interest.

## Ethical approval

The study was registered at the University research system (Code 62713). Approval was obtained from the Ethics committee at Tabriz university of medical sciences (code: IR.IRAN.REC.1398.479, Date: 2019-07-29). Written informed consent was obtained from all participants before recruitment. We adhered to all Helsinki statement principles during the study.

## Authors’ contributions

All of the researchers participated in the conception and design of the study. AS and SA developed the questionnaire. MN and NTA, recruited the participants and collected the data, and MN wrote the manuscript. MN and SMAC, MAJ analyzed the data and interpreted the results. SMAC, SHAA, MAJ, NTA and MM revised and edited the manuscript. All the authors read the ending manuscript and agreed with the content.

**[Table T1]**. 

**Table 1 T1:** Demographic and obstetric characteristics of women (n=200^*^)

**Characteristic**	**n (%)**
Birth place (Alzahra hospital)	146 (73.0)
Educational level	
5-11 years	83 (41.5)
Diploma	71 (35.5)
University	46 (23.0)
Household income sufficiency^a^	
Sufficient	48 (24.1)
Relatively sufficient	107 (53.8)
Not sufficient	44 (22.1)
Parity	
1	87 (43.5)
2	86 (43.0)
3+	27 (13.5)
Unwanted pregnancy	48 (24.0)
Multiple pregnancy^b^	23 (11.5)
Previous preterm birth	39 (20.5)
Admit for major complications during last pregnancy	48 (24.0)
Type of birth	
Vaginal	68 (34.0)
Emergency caesarean section	67 (33.5)
Repeated caesarean section	66 (32.0)
Skin to skin contact at the first^a^ hour after child birth	85 (42.7)
Breastfeeding in the first day^a^	55 (27.6)
Gestational age (weeks)	
Extremely preterm (< 28^0^)	8 (4.0)
Very preterm (28^0^-31^6^)	24 (12.0)
Moderate preterm (32^0^-33^6^)	46 (23.0)
Late preterm (34^0^-36^6^)	122 (61.0)
Mean (SD, range)	33.3 (2.4, 25-36)
Birth attendant	
Obstetrician	30 (15.0)
Obstetric resident	167 (83.5)
Midwife/midwifery student	3 (1.5)
Mean age (SD, range), years	29.3 (6.9, 15-44)

*For one participant, no demographic data was collected. ^a^ One more missing value. ^b^Two were triplets.

**Table 2:** Distributional characteristics of individual Iranian P-BESS items (n=201)

**Item**	**Item content**	Domain*	**Mean**	**SD**	**Range**	**Skew**	**Kurtosis**
PBES1	The staff explained everything really well	Information	4.2	1.0	1-5	-1.37	1.47
PBES 2	There was a pleasant atmosphere in the room	Empathy	4.0	1.0	1-5	-1.03	0.68
PBES 3	The staff made me feel cared for as an individual	Empathy	4.2	0.9	1-5	-1.54	3.04
PBES 4	The staff took control of the situation	Empathy	4.4	0.8	1-5	-1.74	4.23
PBES 5	I was given all the information I needed	Information	4.2	0.9	1-5	-1.30	1.37
PBES 6	The staff put me at ease	Empathy	4.2	0.9	1-5	-1.41	1.99
PBES 7	The staff were encouraging	Empathy	4.1	0.9	1-5	-1.22	1.48
PBES 8	I understood what was happening	Information	4.0	1.0	1-5	-0.86	0.15
PBES 9	The staff were reassuring (R)	Empathy	4.2	0.8	1-5	-1.08	0.98
PBES 10	I did not have confidence in the staff	Confidence	4.1	0.9	1-5	-1.31	2.21
PBES 11	The staff explained to me what would happen during birth	Information	3.7	1.2	1-5	-0.57	-0.71
PBES 12	The staff did not listen to what I had to say (R)	Confidence	3.9	0.9	1-5	-1.12	1.37
PBES 13	The staff kept me informed of what was happening	Information	3.9	1.0	1-5	-0.90	0.21
PBES 14	The staff did not understand how I was feeling (R)	Confidence	3.7	1.1	1-5	-1.03	0.47
PBES 15	The staff explained to me what would happen to my baby when she/he was born	Information	3.6	1.2	1-5	-0.62	-0.60
PBES 16	There were occasions when no one explained to me what was going on (R)	Information	3.7	1.2	1-5	-0.69	-0.52
PBES 17	The staff were warm and friendly	Empathy	4.2	0.9	1-5	-1.51	2.58
PBES 18^a^	I was very satisfied with the care during the birth	--	4.1	0.9	1-5	-1.22	1.47
PBES 19^a^	The care during the birth could have been improved	--	3.1	1.2	1-5	-0.12	-0.92

P-BESS: Preterm birth experiences and satisfaction scale, R: Reversed item, Attainable range score for each item was 1-5. *Domains of the Iranian Preterm Birth Experiences and Satisfaction: Information = Information and Explanations, Empathy = Staff professionalism and Empathy, Confidence = Confidence in Staff. ^a^ These items were used to explore convergent validity.

**Table 3:** Characteristics of Iranian-PBES sub-scales and total score* (n = 201)

**PBES scale**	**Raw score**			**Percentage of the total possible score**			**Skewness**	**Kurtosis**
	**Mean**	**SD**	**Range**	**Mean**	**SD**	**Range**		
Staff professionalism and Empathy (7 items)	29.4	5.1	9-35	80.0	18.3	7.1-100	-1.24	2.02
Information and Explanations (7 items)	27.4	5.4	10-35	72.8	19.3	10.7-100	-0.69	0.19
Confidence in Staff (3 items)	11.7	2.5	4-15	72.5	20.3	8.3-100	-0.97	0.77
Total (17 items)	68.5	11.1	24-85	75.7	16.2	10.3-100	-0.95	1.30

PBES: Preterm Birth Experiences and Satisfaction *For raw score, each item scored from 1 to 5. The higher score, the better experience and satisfaction.

**Table 4:** Factors structure and component loadings of Iranian preterm birth experiences and satisfaction scale

**Items**	**Component loadings**		
	**Factor 1**	**Factor 2**	**Factor 3**
**1. Staff professionalism and Empathy**			
The staff put me at ease	0.88		
The staff made me feel cared for as an individual	0.78		
The staff were warm and friendly	0.75		
The staff were encouraging	0.72		
There was a pleasant atmosphere in the room	0.72		
The staff took control of the situation	0.66		
The staff were reassuring	0.64		
**2. Confidence in Staff**			
The staff did not listen to what I had to say		0.81	
The staff did not understand how I was feeling		0.77	
I did not have confidence in the staff		0.63	
**3. Information and Explanations**			
I was given all the information I needed			0.77
The staff explained everything really well			0.72
The staff kept me informed of what was happening			0.71
The staff explained to me what would happen to my baby when she/he was born			0.60
I understood what was happening			0.58
The staff explained to me what would happen during birth			0.57
There were occasions when no one explained to me what was going on			0.49
**% of total variance explained by the factor**	42.9%	8.0%	4.5%
**% of total variance explained by the scale**		55.4%	
Cronbach’s alpha	0.911	0.808	0.852
	Total Score = 0.922		
Intraclass correlation coefficient (95% CI)	0.85 (0.55, 0.95)	0.92 (0.76, 0.97)	0.83 (0.50, 0.94)
	Total Score = 0.88 (0.63, 0.96)		

P-BESS: Preterm birth experiences and satisfaction scale

**Appendix 1:**